# Supplementary material for: Expression and Functional Role of the P2X7 Receptor in Glioma Models and Tissues: A Systematic Review
Source: Cancers (Basel). 2025 Nov 24;17(23):3748. doi: 10.3390/cancers17233748 (PMC12691461; doi:10.3390/cancers17233748)
Supplement: Supplementary file 1 [file cancers-17-03748-s001.zip › Supplementary Table S1. Inclusion and exclusion criteria applied in the systematic review.docx]

**Supplementary Table S1.** Detailed inclusion and exclusion criteria used to identify eligible studies for this systematic review.

| **Inclusion Criteria** | **Exclusion Criteria** |
| --- | --- |
| Original experimental studies (in vitro or in vivo). | Reviews, meta-analyses, commentaries, letters, or case reports. |
| Studies investigating the expression and/or function of the P2X7 receptor (P2X7R) in glioma. | Studies not investigating the role or expression of P2X7R in glioma. |
| Studies performed on human glioma cell lines, patient-derived glioma cells, or animal glioma models. | Studies focused exclusively on other cancers or diseases not involving glioma models. |
| Full text available in English. | Studies focusing exclusively on bioinformatic data without any experimental validation were excluded. |
